# Maternal and fetal outcomes of patients with liver cirrhosis: a case-control study

**DOI:** 10.1186/s12884-021-03756-y

**Published:** 2021-04-08

**Authors:** Xiang Gao, Yunxia Zhu, Haixia Liu, Hongwei Yu, Ming Wang

**Affiliations:** 1 Department of Gynecologic Oncology, Beijing Obstetrics and Gynecology Hospital, Capital Medical University, Beijing, China; 2 Department of Clinical Care Medicine of Liver Diseases, Beijing Youan Hospital, Capital Medical University, Beijing, China; 3 Department of Obstetrics and Gynecology, Beijing Youan Hospital, Capital Medical University, Qihelou street No.17, Dongcheng District, Beijing, 100006 China

**Keywords:** Liver cirrhosis, Pregnancy, Child-Turcotte-Pugh score, HBV

## Abstract

**Background:**

We aimed to describe the characteristics and outcomes in pregnant women with liver cirrhosis, and identify the predictors of adverse events of mother and fetus.

**Methods:**

Retrospectively collected mothers with liver cirrhosis in our center from 6/2010 to 6/2019. Women without liver cirrhosis were selected as a control in a 1:2 ratio. The primary assessment was the frequency of maternal and fetal adverse events. The secondary assessment was the adverse events in patients continuing pregnancy or not and the factors to predict the severe adverse events.

**Results:**

Of 126 pregnancies enrolled, 29 pregnancies were terminated for worrying disease progression and 97 pregnancies continued. One hundred ninety-four pregnancies without liver cirrhosis were selected as control. At baseline, patients with liver cirrhosis have a lower level of platelet, hemoglobin, prothrombin activity, and a higher level of ALT, total Bilirubin, creatinine. Compared to control, patients with liver cirrhosis had a higher frequency of adverse events, including bleeding gums (7.2%vs. 1.0%), TBA elevation (18.6%vs.3.1%), infection (10.3%vs.0.5%), cesarean section (73.6%vs.49.5%), postpartum hemorrhage (13.8% vs 2.1%), blood transfusion (28.9% vs 2.1%), new ascites or aggravating ascites (6.2% vs.0%), MODS (7.2% vs.0.5%) and intensive care unit admissions (24.1% vs 1.1%). The incidence of severe maternal adverse events was also higher (32.0% vs 1.5%). Women who chose to terminated the pregnancy had less severe adverse events (3.4% vs.32.0%).

A higher frequency of fetal/infants’ complications was observed in liver cirrhosis population than control, including newborn asphyxia (10.2% vs1.1%), low birth weight infant (13.6% vs. 2.6%). In patients who progressed into the third trimester, multivariable regression analysis demonstrated that severe adverse events were associated with a higher CTP score (OR 2.128, 95% CI [1.002, 4.521], *p* = 0.049). Wilson’s disease related liver cirrhosis has a better prognosis (OR = 0.009, 95% CI [0, 0.763], *p* = 0.038).

**Conclusions:**

The incidence of the adverse events was significantly increased in pregnancies complicated by cirrhosis. The predictor of severe adverse events is higher CTP score. Wilson’s disease induced liver cirrhosis have a better prognosis. Timely termination of pregnancy during the first trimester may avoid the incidence of severe adverse events.

**Supplementary Information:**

The online version contains supplementary material available at 10.1186/s12884-021-03756-y.

## Background

Liver cirrhosis is a chronic hepatocyte injury with extensive fibrosis and nodular regeneration [1]. Pregnancy with liver cirrhosis is uncommon due to disturbances in endocrine metabolism, especially estrogen [2, 3]. With improved therapeutic options for liver disease, more women are presenting for prenatal care with concomitant cirrhosis [4].

Pregnant women with advanced cirrhosis are associated with an increased risk of complications such as new-onset or deteriorated ascites for blood volume changes, bleeding from esophageal varices, liver failure, and hepatorenal syndrome [5, 6]. Besides the deteriorate complication of liver cirrhosis, adverse events of mothers and fetuses, such as spontaneous abortion, stillbirth, fetal or neonatal demise, placental abruption, preeclampsia, preterm delivery, small-for-gestational-age neonate, and postpartum hemorrhage are at an increased risk in women with cirrhosis [7].

Limited data exists regarding the negative maternal and fetal outcomes in mothers with liver cirrhosis. Most studies are case series [3, 7–9] and further studies involving larger patient populations are necessary to better guide clinical decision-making, improve prognosis, allow risk stratification, and design clinical trials. Therefore, we conducted a comparative study about the negative maternal outcomes (liver-related adverse events and obstetrical complications) and fetal/infant outcomes. Also, we evaluate the predictors and potential measures to improve the outcomes of the mother and fetus by comparing cirrhosis mothers with and those without adverse events.

## Methods

### Patient selection and study design

This was a retrospective, case–controlled, open-label study that included patients from a single tertiary university medical center, Beijing YouAn Hospital in China, from June 2010 through June 2019. This medical center is the appointed hospital by the department of health in Beijing to evaluate mothers with liver diseases, including pregnant women diagnosed to have liver cirrhosis. The trial was approved by the institutional ethics review committee (approval number: Jing-you-ke-lun-zi (2020)050) and the need for informed consent was obtained.

Case records of patients were reviewed for the following eligibility criteria: age between 20 and 45 years; clinical diagnosed as liver cirrhosis (Ultrasonography, fibroscan, serum marker, or liver biopsy showed stage IV fibrosis, or clinical signs of cirrhotic complications including but not limited to varices, ascites, encephalopathy, hepatorenal syndrome) before or during the pregnancy; delivered in Beijing YouAn hospital institution where both gastrointestinal and prenatal follow-up visits were conducted. Key exclusion criteria: liver diseases without cirrhosis including Wilson disease, activated hepatitis B or C, hemophagocytic lymphohistiocytosis, hepatocellular carcinoma, or cytotoxic drugs. A group of pregnant mothers without liver cirrhosis were randomly selected (in the same admission period) in a 1:2 ratio to serve as the control group.

### Study procedures and data collections

Demographic and clinical data were extracted from the electronic clinical record and paper charts. The data were collected for analysis of baseline information at first trimester: age, gravidity, parity, lab test (hemoglobin, platelet, alanine transaminase (ALT), albumin, total bilirubin, prothrombin activity, and creatinine), and pertinent physical findings. For women with liver cirrhosis, additional data collection was performed including documentation of gestational weeks at the time of the diagnosis, clinical signs, and supplementary examinations, such as ultrasonography, digestive endoscopy and liver puncture biopsy.

### Outcome measurements

The primary outcomes of our study were the frequencies of maternal and fetal/infant adverse events. Negative maternal adverse events includs pregnancy complications, obstetrical complications, and liver-related complications (including ALT elevation (>2ULN), liver failure, deteriorate of symptoms of liver cirrhosis, renal failure, coagulation disorders, shock, infection, and death). The aforementioned outcomes were compared between groups. Pregnancy complications and obstetric complications including hypothyroidism during pregnancy, pregnancy-induced hypertension (PIH), gestational diabetes (GDM), placenta previa, data regarding medications, pregnancy complications, postpartum hemorrhage (> 1000 ml in cesarean section, > 500 ml in vaginal delivery, > 400 ml in abortion) and other obstetric complications were also collected during the delivery or after the delivery. Adverse events of fetal/infant were defined as fetal development restriction, fetal distress, preterm delivery, neonatal ICU admission and asphyxia of newborn (Apgar score 0–7) etc.

Our secondary outcomes were to evaluate the risk of deteriorating symptoms of liver cirrhosis between continuing and discontinuing pregnancy. Also, the roles of predelivery splenectomy or intrauterine balloon in reducing postpartum hemorrhage, hysterectomy, and severe adverse events of mothers were evaluated. Severe adverse events include placenta abruption, postpartum hemorrhage(> 1000 ml), hysterectomy, poor wound healing, infection, MODS, subarachnoid hemorrhage, coagulation disorders, new ascites or aggravating ascites, upper gastrointestinal hemorrhage, fetal death, ALT elevation(>10ULN).

### Statistical analysis

Baseline information of two groups were summarized utilizing descriptive statistics, including percentage and means ±standard deviation (SD). For the quantitative variable, the differences of two group were compared with the Student’s t-test. For categorical variables, the differences of two group were compared with y χ2-test or Fisher’s exact test. Multivariate classification logistic regression was used to adjust variables on predicting adverse events. Adverse events were replaced with 1 and normal outcomes were replaced with 0 during regression. The significance level was set at *P* < 0.05, all data were analyzed by SPSS 23.0 (SPSS, IBM., New York).

## Results

### Study population

**Fig. 1:**
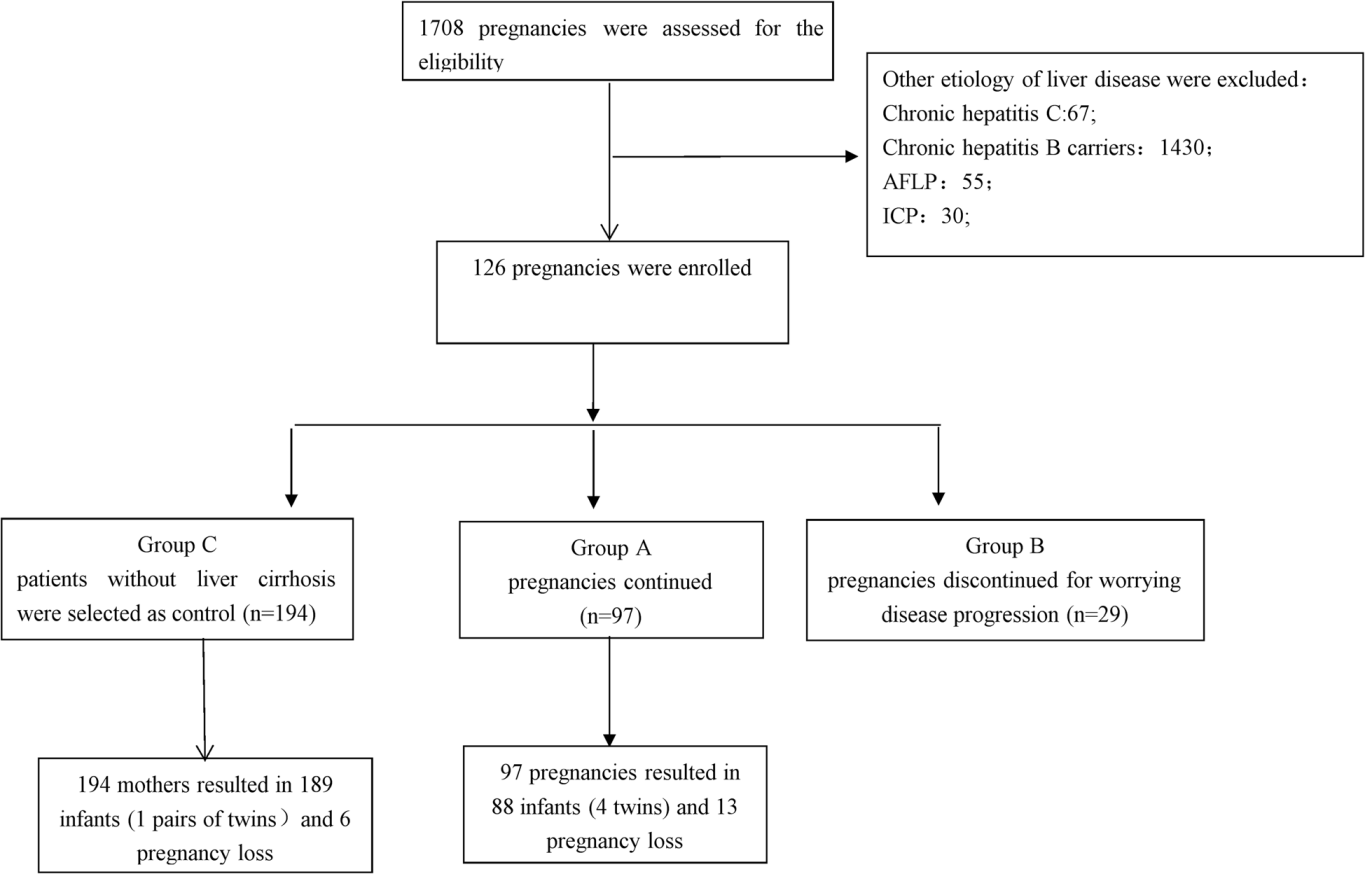
Disposition of Mothers and Infants. AFLP, Acute fatty liver of pregnancy; ICP, intrahepatic cholestasis pregnancy

**Fig. 2:**
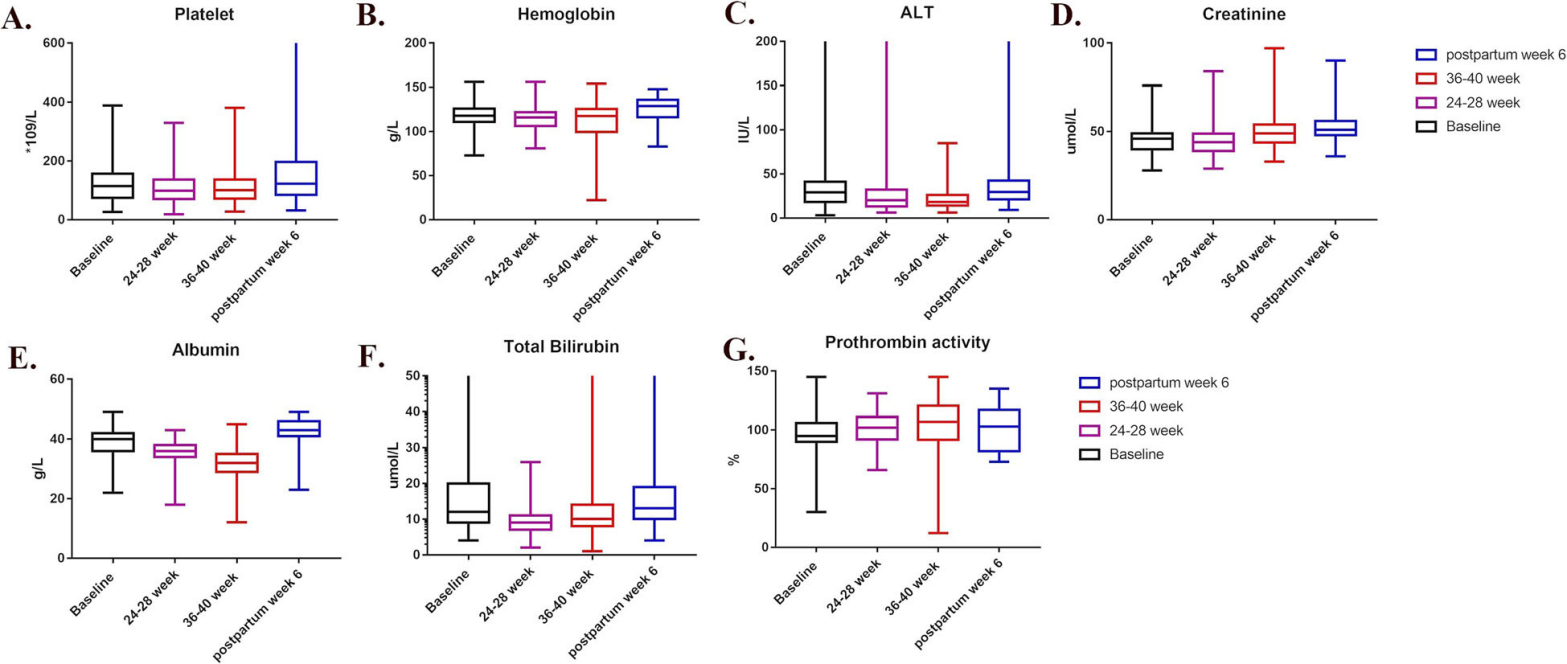
The change of liver biochemistry during the follow-up in the patients with liver cirrhosis. ALT, alanine transaminase

During the enrollment period, the consecutive medical records of 1708 pregnancies at our center were reviewed. Among them, 1582 pregnancies were excluded for not progressing into liver cirrhosis (Fig. 1) and 126 pregnancies were diagnosed with liver cirrhosis before or during pregnancy. Ninety-seven continued pregnancies were enrolled into group A and **29** terminated pregnancies for the concerning of disease progression were enrolled into group B. The group A were matched with 194 patients (1:2 ratio) without liver diseases based on the registration numbers and assigned to group C. Patients who received cesarean section in group A were further assigned into group A1 or group A2 based on whether using intrauterine balloon to prevent postpartum hemorrhage. Patients who were screened and enrolled in the different study groups are shown in Fig. 1**.** The data of liver biochemistry changes during the pregnancy is shown in Fig. 2.

### The outcomes of patients with and without liver cirrhosis

Clinical characteristics of patients at screening in each group are shown in Table 1. When compared to patients without liver cirrhosis (group C), patients with liver cirrhosis have significantly lower level of platelet (123.51 ± 66.66 vs. 239.60 ± 54.70*109/L,*P* = 4.34*10–40), hemoglobin (117.37 ± 14.91 vs. 124.97 ± 14.13 g/L,*P* = 3.7*10–7), prothrombin activity (96.79 ± 17.27 vs. 105.87 ± 11.14%, *p* = 1.0*10–5) and higher level of ALT(43.38 ± 60.49vs.20.74 ± 21.42 IU/L, *p* = 5.98*10–4), Total Bilirubin(20.35 ± 39.31 vs.11.00 ± 6.78 umol/L, *p* = 0.023), and creatinine (45.72 ± 7.49 vs.43.00 ± 6.65 umol/L, *p* = 2.25*10–3). There were no differences of the other baseline values between two groups, including the age (30.79 ± 5.01 vs.31.38 ± 4.13, *p* = 0.322), ratio of primigravida (36.1% vs.40.2%), and multiple pregnancy times (33% vs.48.2%).

**Table 1:** Baseline characteristics of mothers with and without liver cirrhosis (*n* = 291)

	Group A (*n* = 97)	Group C (*n* = 194)	t/χ2/Z, ***P***
**Age (mean ± SD, years)**	30.79 ± 5.01	31.38 ± 4.13	t = 0.993, *p* = 0.322
**Gravidity, n (%)**			
1	35 (36.1)	78 (40.2)	Z = 0.555, *p* = 0.579
2	30 (30.9)	55 (28.4)	
> 2	32 (33)	61 (31.4)	
**Multipara, n (%)**	32 (33)	83 (42.8)	χ2 = 2.595, *P* = 0.107
**Lab on first visit (mean ± SD)**			
Platelet (*10^9^/L)	123.51 ± 66.66	239.60 ± 54.70	t = 15.638, *p* = 4.34*10^− 40^
Hemoglobin(g/L)	117.37 ± 14.91	124.97 ± 14.13	t = 4.191, *p* = 3.7*10^−7^
ALT (IU/L)	43.38 ± 60.49	20.74 ± 21.42	t = 3.539, *p* = 5.98*10^− 4^
Albumin(g/L)	39.49 ± 5.22	43.85 ± 27.17	t = 1.549, *p* = 0.123
Total Bilirubin (umol/L)	20.35 ± 39.31	11.00 ± 6.78	t = 2.302, *p* = 0.023
Prothrombin activity (%)	96.79 ± 17.27	105.87 ± 11.14	t = 4.579, *p* = 1.0*10^−5^
Creatinine (umol/L)	45.72 ± 7.49	43.00 ± 6.65	t = 3.084, *p* = 2.25*10^−3^

*ALT* Alanine aminotransferase

As is shown in Table 2, a significantly higher frequency of adverse events (71.1% [69/97] vs12.9% [25/194], *p* < 0.05) was observed in group A than group C. In terms of obstetrical complications, higher incidence of cesarean section (73.6%vs.49.5%, *P* < 0.05), postpartum hemorrhage (13.8% vs 2.1%, *p* < 0.05) and blood transfusion (28.9% vs 2.1%, *p* < 0.05) were observed in group A than group C. Most mothers who had cesarean sections in group A had the following top three indications: liver cirrhosis related disease, cesarean section performed on previous delivery, and fetal distress during labor. Most mothers who had cesarean sections in group C had the following top three indications: cesarean section performed on previous delivery, failure to progress from the first to the second stage of labor, and fetal distress during labor.

**Table 2:** Maternal and fetal/infant adverse events of in mothers with and without liver cirrhosis(*n* = 291)

	Group A	Group C	t/χ2, ***P***-value
**Maternal Complications, n (%)**	**(*n*** **= 97)**	**(*n*** **= 194)**	
**First trimester**	*n* = 97	*n* = 194	
Ectopic pregnancy	3 (3.1)	0 (0)	χ2# = 3.41, *P* = 0.065
Induced abortion	1 (1)	0 (0)	P** = 0.33
Missed abortion	4 (4.1)	6 (3.1)	χ2# = 0.013, *P* = 0.91
Subclinical hypothyroidism during pregnancy	7 (7.2)	6 (3.1)	χ2# = 1.71, *P* = 0.19
**Second trimester**	***n*** **= 89**	***n*** **= 188**	
Spontaneous abortion	2 (2.2)	0 (0)	P** = 0.10
Pregnancy-induced hypertension	8 (8.2)	5 (2.7)	χ2# = 3.39, *P* = 0.065
**Third trimester**	***n*** **= 87**	***n*** **= 188**	
Gestational diabetes mellitus	18 (20.7)	27 (14.4)	χ2 = 1.74, *P* = 0.19
Intrauterine fetal death	3 (3.4)	0 (0)	P** = 0.031
Oligohydramnios	1 (1.1)	12 (6.4)	χ2 = 3.62, *P* = 0.057
Placenta Previa	1 + 3 (4.6)	2 (1.1)	χ2# = 2.02, *P* = 0.16
Placenta abruption	1 (1.1)	1 (0.5)	P** = 0.55
Cesarean section	64 (73.6)	93 (49.5)	χ2 = 14.10, *P* = 1.7*10^−5^
**Postpartum**	***n*** **= 87**	***n*** **= 188**	
Postpartum Hemorrhage	12 (13.8)	4 (2.1)	χ2 = 14.77, *P* = 1.2*10^−4^
Intensive care unit admission	21 (24.1)	2 (1.1)	χ2 = 41.32, *P* = 1.3*10^−10^
Intrauterine balloon pressure	15 (17.2)	7 (3.7)	χ2 = 14.77, *P* = 1.2*10^−4^
Hysterectomy	1 (1)	0 (0)	P** = 0.33
Poor wound healing	3 (3.4)	1 (0.5)	χ2# = 1.79, *p* = 0.18
**Complication due to liver-related events**	*n* = 97	*n* = 194	
Bleeding Gums	7 (7.2)	2 (1.0)	χ2# = 6.32, *p* = 0.012
Infection	10 (10.3)	1 (0.5)	χ2 = 14.47, *p* = 1.4*10^−4^
**MODS**	7 (7.2)	1 (0.5)	χ2# = 8.50, *p* = 4.0*10^−3^
Right heart failure	1 (1.0)	0 (0)	p** = 0.33
Respiratory failure	1 (1.0)	0 (0)	p** = 0.33
Acute liver failure	2 (2.1)	0 (0)	p** = 0.11
Renal insufficiency	3 (3.1)	1 (0.5)	χ2# = 1.55, *p* = 0.21
**Subarachnoid hemorrhage**	1 (1.0)	0 (0)	p** = 0.33
**Coagulation disorders**	2 (2.1)	1 (0.5)	χ2# = 0.38, *p* = 0.54
**New ascites or aggravating ascites**	6 (6.2)	0 (0)	χ2# = 9.38, *p* = 2.0*10^−3^
**Upper gastrointestinal hemorrhage**	2 (2.1)	0 (0)	p** = 0.11
**TBA elevation**	18 (18.6)	6 (3.1)	χ2 = 20.43, *p* = 6.0*10^−6^
**ALT elevation**			
Mild	16 (16.5)	4 (2.1)	Z = 6.898, *p* = 5.3*10^−12^
Moderate	13 (13.4)	2 (1.0)	
Severe	2 (2.1)	0 (0)	
**Blood Transfusion**	28 (28.9)	4 (2.1)	χ2 = 47.47, *p* = 5.6*10^−12^
**Severe adverse events**	31 (32.0)	3 (1.5)	χ2# = 57.964, *p* = 2.7*10^−14^
**Fetal/newborn complications,** n (%)	**(*n*** **= 88)**	**(*n*** **= 189)**	**t/χ2,** ***P*-value**
**Gestational weeks of delivery (mean ± SD, weeks)**	37.62 **± 2**.63	39.07 **±** 1.53	t = 4.75, *p* = 6.0*10^−6^
Fetal development restriction	1 (1.1)	2 (1.1)	χ2# = 0, *p* = 1
Low birth weight infant	12 (13.6)	5 (2.6)	χ2 = 12.59, *p* = 3.9*10^−4^
Fetal macrosomia	8 (9.1)	10 (5.3)	χ2 = 1.43, *p* = 0.23
Fetal distress	4 (4.5)	4 (2.1)	χ2# = 0.55, *p* = 0.46
Preterm delivery	27 (30.7)	8 (4.2)	χ2 = 38.05, *p* = 6.9*10^−10^
Asphyxia of newborn	9 (10.2)	2 (1.1)	χ2 = 10.94, *p* = 9.4*10^−4^
Apgar score 4–7 at 1 min	4	1	
Apgar score 0–3 at 1 min	5	1	
Neonatal ICU admission	8 (9.1)	3 (1.6)	χ2# = 7.01, *p* = 8.0*10^−3^
Fetal/newborn death	4 (4.5)	0 (0)	χ2 = 5.82, *p* = 0.016

*ALT* Alanine aminotransferase; *TBA* Total bile acid; *MODS* Multiple organ dysfunction; *ICU* Intensive care unit. #continuous correction; ** Fisher’s test

In Group A, 17.2% (15/87) patients received intrauterine balloon pressure and 1 patient received subtotal hysterectomy to prevent further postpartum hemorrhage. In group C, only 3.7% (7/88) patients received intrauterine balloon pressure and no one underwent hysterectomy.

Other obstetrical outcomes, for example, more ectopic pregnancy (3.1% vs. 0%, *p* = 0.065), pregnancy-induced hypertension (8.2 vs.2.7, *p* = 0.065), gestational diabetes mellitus (20.7%vs. 14.4%, *P* = 0.187), placenta previa (4.6% vs. 1.1%, *p* = 0.155), poor wound healing (3.4% vs. 0.5%, *p* = 0.181) and less oligohydramnios (1.1% vs.6.4%, *p* = 0.057) seemed to be occurred in group A than group C, however no statistical significance was found.

In terms of liver-related disease, higher rates of bleeding gums (7.2%vs. 1.0%, *p* < 0.05), TBA elevation (18.6%vs.3.1%, *P* < 0.05), new ascites or aggravating ascites (6.2% vs.0%, *p* < 0.05), MODS (7.2% vs.0.5%, *p* < 0.05) and intensive care unit admissions (24.1% vs 1.1%, *p* < 0.05) were found in group A than group C. 10.3% infection (4 bacterial peritonitis, 1 chorioamnionitis, 1 fungi infection, 3 severe pneumonia and 1 intestinal infections) were observed in group A. However, only 1 case with upper tract infection were observed in group C (*p* < 0.05). There were no cases of maternal deaths but 2 cases of variceal bleeding in our study. One case was a tubal ectopic pregnancy and received laparoscopic salpingectomy. One day later, she had variceal bleeding more than 1000 ml. The other case suffered from variceal bleeding during postpartum. Eight patients were diagnosed with esophageal varices before the third trimester and 5 patients received endoscopic treatment or pericardial devascularization before delivery. One patient developed progressive jaundice at 8 weeks, and progressive disturbance of consciousness after a fall at 22 weeks, MRI indicated subdural hematoma and subarachnoid hemorrhage. She recovered and successfully delivered after treatment.

In terms of fetal/infant complications, a significantly higher frequency of preterm delivery (30.7% vs. 4.2%, *p* = 6.89*10^− 10^), low birth weight infant (13.6% vs. 2.6%, *p* = 3.88*10^− 4^), asphyxia of newborn (10.2% vs. 1.1%, *p* = 9.42*10^− 4^), neonatal ICU admission (9.1% vs. 1.6%, *p* = 0.008) and fetal/newborn death (4.5% vs.0%, *p* = 0.016) in group A than in group C. The intrauterine fetal death were occurred at 31.29 ± 6.02 weeks.

### The outcomes of patients with liver cirrhosis continuing and terminating pregnancy

The clinical characteristics of the liver cirrhosis patients whose pregnancy was continued or terminated were summarized in suppl Table 1. Compared to patients who chose to continue the pregnancy, patients who discontinued the pregnancy for concerning the disease deteriorated had more history of gravidity (89.6% vs. 63.9%) and multipara (79.3% vs. 33%). More women in group B had hypersplenism (thrombocytopenia or anemia) than group B (54.6%vs.79.3%, *p* = 0.017). The lower level of PLT (85.34 ± 54.49 vs. 123.52 ± 66.66, *p* = 0.006), PTA (89.56 ± 11.50 vs.96.79 ± 17.27, *p* = 0.036) and a higher level of Albumin (41.81 ± 5.00 vs.39.49 ± 5.22, p = 0.036) were found in group B than group A. Compared to women who chose to discontinue the pregnancy, women in group B had more severe adverse events (32.0 vs.3.4, *p* = 0.002).

### The predictors of severe adverse events in patients who had liver cirrhosis and persisted into third trimester

To investigate the predictors of severe adverse events in this population, we performed multivariate classification logistic regression analysis to compare the baseline variables. Patients with severe adverse events had a higher CTP scores (OR = 2.128, 95% CI:[1.002, 4.521],*p* = 0.049). Besides, patients with liver cirrhosis caused by Wilson’s disease had a better prognosis than by HBV infection (OR = 0.009, 95% CI: [0, 0.763], *p* = 0.038). No other predictors of negative maternal outcomes were found with a significant difference in baseline values (suppl Table 2). In total, 79 of 126 pregnancies were with known liver cirrhosis prior to pregnancy and the others were diagnosed during pregnancy. Thirty-nine of them received screening endoscopy prior to pregnancy and 13 were found with varices. No patients underwent screening endoscopy during the pregnancy. Fifty-nine of 126 pregnancies had platelet < 100*10^9^/L in the baseline, and 6 of 59 patients were with known varices. Three of the 6 patients selected to terminatethe pregnancy in early stage forconcerning progression of disease.threepatients continued the pregnancy had no adverse events.

## Discussion

Pregnancy and liver cirrhosis are a high-risk combination. The risks for the mother and the fetus are associated with worsening of liver decompensation and progression of portal hypertension: ascites, hepatorenal syndrome, hepatic encephalopathy, and variceal hemorrhage [8]. Though most of our patients were in the compensatory stage, patients with liver cirrhosis have a significantly lower level of platelet, hemoglobin, prothrombin activity, and a higher level of ALT, total bilirubin, and creatinine. The incidence of spontaneous abortions and abnormal uterine bleeding were increased as the progression of liver cirrhosis [10]. However, no difference in missed abortion and spontaneous abortion were found between pregnant patients with cirrhosis or not in a previous larger population study [5] and in our study.

The maternal death rates had decreased from 10 to 1% ~ 1.8% as the medical therapy developing [3, 11, 12]. In the previous study, most maternal mortality was attributed to hemorrhage from gastrointestinal varices, occurring most commonly in the second trimester and during labor [9, 13]. There were no cases of maternal deaths but 2 cases of variceal bleeding in our study. One of them might be caused by laparoscopic pneumoperitoneum and stress of surgery. However, rate of obstetric complications were still 61% in women with cirrhosis, which was higher than 12% in the control group [7]. In the previous study with 103 pregnancies women with cirrhosis there were 12 hospitalizations during pregnancy due to liver-related events, including one case with bleeding esophageal varices. The risk of caesarean delivery (36% versus 16%), low birth weight (15% versus 3%), and preterm delivery (19% versus 5%) were higher than the common population [5]. In our study, liver cirrhosis has a higher frequency of severe adverse events (32.0% vs. 1.5%) than control. The most common adverse events in our study were bleeding gums, TBA elevation, cesarean section, postpartum hemorrhage, new ascites, or aggravating ascites and MODS. 24.1% patients needed intensive care unit admissions and 28.9% patients needed blood transfusion. Except for these risks, we found more subclinical hypothyroidism during pregnancy (7.2% vs. 3.1%), pregnancy-induced hypertension (8.2% vs.2.7%), GDM (20.7%vs. 14.4%) seemed to occur in patients with liver cirrhosis which might be associated with the poor liver metabolism. However, no statistically significant differences were found. Besides, patients with liver cirrhosis had more placenta previa (4.6% vs. 1.1%), and less oligohydramnios (1.1% vs.6.4%) which were related to more preterm delivery (30.7% vs. 4.2%). More TBA elevation (18.6% vs. 3.1%) or preterm delivery might be associate with the higher asphyxia of newborn (10.2% vs. 1.1%) and fetal/newborn death (4.5% vs.0%) in the liver cirrhosis patients.

The main etiological causes of cirrhosis in this study were viral hepatitis which accounted for 86.6% of all diagnoses. This differs from previous studies in which alcoholic liver disease was the main underlying cause of liver disease. This was likely reflects a difference in the prevalence of liver disease in these populations [8, 14, 15]. In our study, patients with liver cirrhosis caused by Wilson’s disease had a better prognosis than by HBV infection. Patients with severe adverse events had a higher CTP score. Other factors such as age, the duration of disease, history of delivery, and portal hypertension, and mode of delivery did not predict the severe adverse events well independently. Compared to women who continue the pregnancy, women who chose to discontinue the pregnancy in the first trimester could avoid the most severe adverse events. Then, the management of those patients should be individual. The patients with a higher CTP score should be advised to discontinue the pregnancy in the first trimester.

Our study describes the characteristics and outcomes in pregnant women with liver cirrhosis, and identify CTP score as the predictors of severe adverse events. However, we did not test whether meld score, MELD sodium or ALBI and APRI have predictive value in patients with cirrhosis because serum albumin and creatinine fluctuated during pregnancy. Westbrook et al. and Gonsal korala et al. found ALBI and APRI scores can prognosticate pregnancy outcomes in women with chronic liver disease [16]. However, only 80 pregnancies of them had liver cirrhosis and the receiver operating characteristic for these 2 measures were 0.74 and 0.70, respectively. During the pregnancy, the blood volume increased 30–50%. The concentration of serum albumin and creatinine was lower than the common population. Also, MELD sodium and ALBI were affected by the creatinine and albumin in pregnant population. Therefore, we selected CTP score to evaluate the severity of pregnant patients with liver cirrhosis. Further studies are needed to evaluate the predictive value of these models in pregnant patients. Besides, for the sample size confined, the effect on some pregnancy-related disease is not clear, such as hypothyroidism, GDM, and PIH. It may also be a limitation that it is a retrospective study. A further prospective study is needed to verify the conclusion in this study.

## Conclusion

In this study, we found that the frequency of the severe adverse events (including liver-related complications, obstetrical complications, and fetal or neonatal death) was significantly increased in pregnancies complicated by cirrhosis. The predictor of severe adverse events is higher CTP score. Wilson’s disease induced liver cirrhosis have a better prognosis. Timely terminationof pregnancy during the first trimester may avoid the incidence of those severe adverse events.

## Supplementary Information

**Additional file 1: Suppl Table 1**. Outcomes of mothers with liver cirrhosis continue the pregnancy or not (*n* = 10)

**Additional file 2: Suppl Table 2**. Predictors of severe adverse events in mothers of third trimester (*n* = 87)

## Data Availability

The datasets used and/or analyzed during the current study are available from the corresponding author on reasonable request.

## Abbreviations

CTP score: Child-turcotte-pugh score
HBV: Hepatitis B virus
ALT: Alanine aminotransferase
TBA: Total bile acid
ULN: Upper limit normal
PIH: Pregnancy-induced hypertension
GDM: Gestational diabetes
MODS: Multiple organ dysfunction
ICU: Intensive care unit
AFLP: Acute fatty liver of pregnancy
ICP: Intrahepatic cholestasis pregnancy
