# Correction to: Maternal and fetal outcomes of patients with liver cirrhosis: a case-control study

**DOI:** 10.1186/s12884-021-03823-4

**Published:** 2021-04-24

**Authors:** Xiang Gao, Yunxia Zhu, Haixia Liu, Hongwei Yu, Ming Wang

**Affiliations:** 1 Department of Obstetrics and Gynecology, Beijing Youan Hospital, Capital Medical University, Beijing, China; 2 Department of Clinical Care Medicine of Liver Diseases, Beijing Youan Hospital, Capital Medical University, Beijing, China; 3 Department of Gynecologic Oncology, Beijing Obstetrics and Gynecology Hospital, Capital Medical University, Qihelou street No.17, Dongcheng District, Beijing, 100006 China

**Correction to: BMC Pregnancy Childbirth 21, 280 (2021)**

**https://doi.org/10.1186/s12884-021-03756-y**

Following publication of the original article [1], the authors reported authors were assigned to incorrect affiliations. Correct affiliations are as below.

Dr. Gao Xiang and Yunxia Zhu are in the Department of Obstetrics and Gynecology, Beijing Youan Hospital, Capital Medical University, Beijing, China.

Ming Wang is in the Department of Gynecologic Oncology, Beijing Obstetrics and Gynecology Hospital, Capital Medical University, Qihelou street No.17, Dongcheng District, Beijing, 100006, China.

The original article [1] has been updated.
