# Supplementary material for: Maternal and fetal outcomes of patients with liver cirrhosis: a case-control study
Source: BMC Pregnancy Childbirth. 2021 Apr 8;21:280. doi: 10.1186/s12884-021-03756-y (PMC8033723; doi:10.1186/s12884-021-03756-y)
Supplement: Supplementary file 1 — Additional file 1: Suppl Table 1. Outcomes of mothers with liver cirrhosis continue the pregnancy or not (n = 10) [file 12884_2021_3756_MOESM1_ESM.doc]

**Suppl Table 1. Outcomes of mothers with liver cirrhosis continue the pregnancy or not (n=10)**

| **Baseline values** | **Group A**  **(n=97)** | **Group B**  **(n=29)** | **t/χ^2^, P** |
| --- | --- | --- | --- |
| **Age at the time pregnancy (y)** | **30.79±5.01** | **31.24±5.02** | **t=0.43, p=0.672** |
| **Duration of cirrhosis diagnosed (y)** | **2.02±3.38** | **2.23±2.18** | **t=0.32, p=0.752** |
| **Duration of primary disease (years)** | **7.94±5.99** | **8.70±5.35** | **t=0.61, p=0.545** |
| **Gravidity, n (%)** |  |  |  |
| 1 | 35(36.1) | **3**(10.3) | **Z=3.30, p=0.001** |
| 2 | 30(30.9) | **7**(24.1) |  |
| >2 | 32(33.0) | **19**(65.5) |
| **Multipara, n (%)** | 32(33.0) | **23**(79.3) | χ2=19.48, P=1.0*10^-5^ |
| **CTP score** | **5.70±1.16** | **5.41±0.98** | **t=1.37，p=0.376** |
| **Cause of cirrhosis** | | | |
| HBV infection | **84**(86.6) | **26**(89.7) | **Z=0.37, p=0.711** |
| PBC/AIH | **5**(5.2) | **0**(0) |
| Wilson disease | **1**(1.0) | **1**(3.4) |
| Drug injury | **1**(1.0) | **0**(0) |
| unknown | **6**(6.2) | **2**(6.9) |
| **Drug treatment prior to pregnancy** | | |  |
| **Lab on first visit** | | | |
| Platelet (*109/L) | **123.52±66.66** | **85.34±54.49** | **t=2.81，p=0.006** |
| Hemoglobin(g/L) | **117.37±14.91** | **120.24±13.24** | **t=0.93, p=0.354** |
| ALT(IU/L) | **43.38±60.49** | **34.49±40.34** | **t=0.74, p=0.460** |
| Albumin(g/L) | **39.49±5.22** | **41.81±5.00** | **t=2.12, p=0.036** |
| Total Bilirubin(umol/L) | **20.35±39.31** | **20.56±8.53** | **t=0.027, p=0.978** |
| Prothrombin activity (%) | **96.79±17.27** | **89.56±11.50** | **t=2.12, p=0.036** |
| Creatinine(umol/L) | **45.72±7.49** | **47.62±8.38** | **t=1.16, p=0.248** |
| **Complication before pregnancy** | | | |
| **Portal hypertension** | 79(81.4) | 26(89.7) | χ2=1.08, P=0.298 |
| Splenomegaly | 42(43.3) | 9(31.0) | χ2=1.39, P=0.238 |
| Hypersplenism  (thrombocytopenia or anemia) | 53(54.6) | 23(79.3) | χ2=5.68, P=0.017 |
| Splenectomy or embolism | 9(9.3) | 2(6.9) | Χ2#=0.01, P=0.981 |
| Esophageal varices | 8(8.2) | 5(17.2) | χ2#=1.10, P=0.294 |
| History of Endoscopic treatment  or pericardial devascularization | 5(5.2) | 1(3.4) | χ2#=0, P=1 |
| History of UGIB | 5(5.2) | 5(17.2) | χ2#=2.96, P=0.085 |
| Ascites | 13(13.4) | 3(10.3) | χ2#=0.013, P=0.908 |
| **Liver-related maternal complications** | **Group A**  **(n=97)** | **Group B**  **(n=29)** | **t/χ^2^, P** |
| **Severe adverse events** | 31(32) | **1(3.4)** | χ2=9.578, P=0.002 |
| **Postpartum/post abortion Hemorrhage** | 12(12.4) | **3(10.3)** | χ2#=0, p=1 |
| **Bleeding Gums** | 7(7.2) | **2**(6.9) | χ2#=0, p=1 |
| **Infection** | 10(10.3) | **1**(3.4) | χ2#=0.60, p=0.44 |
| **MODS** | 7(7.2) | **2(6.9)** | χ2#=0, p=1 |
| Right heart failure | 1(1.0) | **1** (3.4) |  |
| Respiratory failure | 1(1.0) | **0**(0) |
| Acute liver failure | 2(2.1) | 1(3.4) |
| Renal insufficiency | 3(3.1) | **0**(0) |
| **Subarachnoid hemorrhage** | 1(1.0) | **0**(0) | P**=1 |
| **Coagulation disorders** | 2(2.1) | **1**(3.4) | P**=0.57 |
| **New ascites or aggravating ascites** | 6(6.2) | **1**(3.4) | χ2#=0.011, P=0.92 |
| **[Upper gastrointestinal hemorrhage](http://dict.youdao.com/w/upper gastrointestinal hemorrhage/" \l "keyfrom=E2Ctranslation)** | 2(2.1) | **0(0 )** | P**=1 |
| **TBA elevation** | 18(18.6) | **3**(10.3) | χ2#=0.57, p=0.45 |
| **ALT elevation** |  | | |
| Mild | 16(16.5) | **4**(13.8) | Z=0.72, p=0.47 |
| Moderate | 13(13.4) | **3**(10.3) |
| Severe | 2(2.1) | **0**(0) |
| **Blood Transfusion** | 28(28.9) | **4**(13.8) | χ2=2.68, p=0.102 |

**CTP Score,** **Child-Turcotte-Pugh Score; ALT, alanine aminotransferase; UGIB, upper gastrointestinal bleeding; TBA, total bile acid; MODS, multiple organ dysfunction; ICU, intensive care unit. #continuous correction; ** Fisher’s test.**
