# Supplementary material for: Maternal and fetal outcomes of patients with liver cirrhosis: a case-control study
Source: BMC Pregnancy Childbirth. 2021 Apr 8;21:280. doi: 10.1186/s12884-021-03756-y (PMC8033723; doi:10.1186/s12884-021-03756-y)
Supplement: Supplementary file 2 — Additional file 2: Suppl Table 2. Predictors of severe adverse events in mothers of third trimester (n = 87) [file 12884_2021_3756_MOESM2_ESM.doc]

**Suppl Table 2. Predictors of severe adverse events in mothers of third trimester (n=87)**

**CTP Score,** **^Child-Turcotte-Pugh Score.^**

| **Variable** | **OR** | **95%CI** | **P-value** |
| --- | --- | --- | --- |
| **Age** | 1.07 | 0.92, 1.23 | 0.40 |
| **Etiology** |  |  |  |
| HBV infection | - |  |  |
| PBC/AIH | 0.053 | 0.002, 1.31 | 0.073 |
| Wilson disease | **9.0*10^-3^** | **0, 0.76** | **0.038** |
| Drug injury | 4.34*10^8^ | - | 1.0 |
| unknown | 1.02*10^9^ | - | 1.0 |
| **Duration of primary disease(years)** | 1.02 | 0.90, 1.15 | 0.81 |
| **Duration of cirrhosis diagnosed (years)** | 0.93 | 0.66,1.32 | 0.69 |
| **Multiple delivery** | 1.92 | 0.37, 9.99 | 0.44 |
| **CTP scores** | **2.13** | **1.00, 4.52** | **0.049** |
| **Hypersplenism** | 0.64 | 0.15, 2.80 | 0.56 |
| **Splenomegaly** | 0.56 | 0.13, 2.45 | 0.44 |
| **Esophageal varices** | 3.94 | 0.097, 160.65 | 0.47 |
| **Ascites** | 0.11 | 0.012,1.04 | 0.054 |
| **mode of delivery** | 3.34 | 0.71,15.68 | 0.13 |
